# Alteration in Resting-State EEG Microstates Following 24 Hours of Total Sleep Deprivation in Healthy Young Male Subjects

**DOI:** 10.3389/fnhum.2021.636252

**Published:** 2021-04-12

**Authors:** Ming Ke, Jianpan Li, Lubin Wang

**Affiliations:** ^1^College of Computer and Communication, Lanzhou University of Technology, Gansu, China; ^2^Institute of Military Cognition and Brain Sciences, Academy of Military Medical Sciences, Beijing, China

**Keywords:** sleep deprivation, resting states, electroencephalography, topographical analysis, EEG microstate

## Abstract

**Purpose:** The cognitive effects of total sleep deprivation (TSD) on the brain remain poorly understood. Electroencephalography (EEG) is a very useful tool for detecting spontaneous brain activity in the resting state. Quasi-stable electrical distributions, known as microstates, carry useful information about the dynamics of large-scale brain networks. In this study, microstate analysis was used to study changes in brain activity after 24 h of total sleep deprivation.

**Participants and Methods:** Twenty-seven healthy volunteers were recruited and underwent EEG scans before and after 24 h of TSD. Microstate analysis was applied, and six microstate classes (A–F) were identified. Topographies and temporal parameters of the microstates were compared between the rested wakefulness (RW) and TSD conditions.

**Results:** Microstate class A (a right-anterior to left-posterior orientation of the mapped field) showed lower global explained variance (GEV), frequency of occurrence, and time coverage in TSD than RW, whereas microstate class D (a fronto-central extreme location of the mapped field) displayed higher GEV, frequency of occurrence, and time coverage in TSD compared to RW. Moreover, subjective sleepiness was significantly negatively correlated with the microstate parameters of class A and positively correlated with the microstate parameters of class D. Transition analysis revealed that class B exhibited a higher probability of transition than did classes D and F in TSD compared to RW.

**Conclusion:** The observation suggests alterations of the dynamic brain-state properties of TSD in healthy young male subjects, which may serve as system-level neural underpinnings for cognitive declines in sleep-deprived subjects.

## Introduction

Sleep is essential for the brain to recover and keep it functioning optimally (Koenis et al., 2013). Sleep deprivation (SD) interferes with the normal functioning of the human brain and even impairs brain function, usually resulting in reduced response times, reduced alertness, and increased perceptual and cognitive distortions (Pilcher and Huffcutt, 1996). Functional magnetic resonance imaging (fMRI) studies have shown that functional connectivity within the default mode network (DMN) and anti-correlation between the DMN and anti-correlated network (ACN) were reduced after sleep deprivation (De Havas et al., 2012; Wang et al., 2015). Graph theory was used to analyze fMRI data in resting state of sleep deprivation to evaluate changes in the brain network structure after sleep deprivation. These studies suggested that sleep deprivation severely impaired the topological properties of the brain’s small-world network (Koenis et al., 2013; Jiang et al., 2018). Previous studies mainly focused on the effects of SD on the topology of the resting-state network and did not specifically study the temporal dynamics of the resting-state network. Compared with fMRI, electroencephalography (EEG) has high temporal resolution, which is beneficial to detecting and recording the temporal dynamics of brain activity. Many methods have been proposed to extract neurophysiologic-relevant features from the recording (Damoiseaux et al., 2006; Mantini et al., 2007). One approach was to use the recorded oscillation characteristics to define the “state” of the signal over time. In this method, brain activity was described by state characteristics, such as the frequency of occurrence or the duration of certain states (Brunet et al., 2011).

EEG microstate analysis is a very mature technique to studying the resting state of the brain. It regards the multichannel EEG signals as a series of quasi-steady microstate sequences, each of which is the electrical potential topological structure at a certain time or time period. The electric field topology at the local maximum of the global field power (GFP) is considered to be the discrete state of the EEG (Lehmann and Skrandies, 1980). The method could simultaneously consider signals recorded in all areas of the scalp. Therefore, it could be used to assess large-scale brain network damage caused by certain psychiatric disorders. In microstate analysis of the resting state of the brain, it was observed that several specific brain states dominated the whole brain activity. Each state remained stable for about 80–120 ms, and then quickly switched to another state (Lehmann and Skrandies, 1980). The duration of EEG microstates is consistent with the time range of spontaneous neural activity. Their different topographies are associated with different types of spontaneity (König et al., 1998) and stimulation-driven psychological processes (Britz et al., 2009). The EEG microstates are correlated with the fMRI resting-state networks (RSNs) (Britz et al., 2010). Spontaneous EEG microstate analysis has been used to assess changes in global brain coordination associated with brain maturation (Pascual-Marqui et al., 1995; Koenig et al., 2002; Lehmann et al., 2010). Microstate changes have also been reported in some psychiatric disorders. Numerous studies have repeatedly reported changes in EEG microstates in patients with schizophrenic disorders (Koenig et al., 1999; Strelets et al., 2003; Kikuchi et al., 2007). In Alzheimer’s, abnormalities in microstate time parameters have been reported (Dierks et al., 1997; Custo et al., 2017). In addition, Strik et al. (1995) found that the duration of microstates in the depressed group had a tendency to become shorter. However, as far as we know, there is no report on spontaneous EEG microstates in SD.

Previous neuroimaging studies have consistently revealed that SD is associated with abnormal competition between large-scale brain networks (Ma et al., 2015; Krause et al., 2017). In particular, three higher-order networks, the so-called salience network (SN), executive control network (ECN), and default mode network (DMN), have received particular attention for their potential relevance to cognition. A large number of studies have shown that SN plays a critical and causal node in the initiating network switching resulting in the participation of the ECN and the non-participation of the DMN (Uddin and Menon, 2009; Menon and Uddin, 2010). The abnormal interaction between these networks is a feature of many mental diseases (Uddin and Menon, 2009; Menon and Uddin, 2010; Zhang et al., 2017). Consistent with this finding, the SN demonstrates reduced activity during the performance of attention tasks following sleep loss (Ma et al., 2015). In our previous work, we have found that the abnormal competition between SN and DMN was significantly correlated with both subjective sleepiness and working memory performance, which may be related to the instability of the awake state (Lei et al., 2015).

Wake-state instability is proposed to account for the more variable behavioral performance after SD, which is driven by the competition between staying alert and falling asleep (Doran et al., 2001). This hypothesis is supported by recent dynamic functional connectivity (dFC) studies that can capture moment-to-moment brain activity variability in SD (Lombardo et al., 2020). Previous studies have reported connectivity states of specific brain networks related to vigilance (the high arousal state and the low arousal state), indicating that the dynamics of these states in SD were related to the temporal fluctuations of vigilance during rest and auditory vigilance tasks (Wang et al., 2016; Patanaik et al., 2018). El-Baba et al. (2019) found that the transition dynamics between functional connectivity (FC) states were destroyed after SD. The change of state transitions could predict the decline rate of the speed of processing after SD (Patanaik et al., 2018). By using resting-state fMRI data, Xu et al. (2018) identified seven SD- and rested wakefulness (RW)-dominant functional connectivity states that exhibited different occurrence probabilities and dwell times across sleep conditions. Moreover, Teng et al. (2019) found that the occurrence of specific brain networks can be used as an index of arousal to track changes in vigilance following SD. Compared with dFC analysis using fMRI data, EEG microstate analysis has high temporal resolution, which is more sensitive in detecting the characterizations of bottom-up and top-down attention control and rapid transitions between quasi-stable brain states.

In this study, we hypothesized that brain resting-state networks would be disrupted after total sleep deprivation (TSD). EEG microstate analysis was applied in this study, aiming to explore the effects of sleep deprivation on brain cognition by revealing differences in dynamic brain activities between the RW and TSD conditions.

## Materials and Methods

### Participants

Twenty-seven healthy volunteers (all males; mean age = 23.3 years, *SD* = 1.9, range = 21–25) participated in the study. All subjects met the inclusion criteria: (1) the sleeping habits were good (sleeping time per day was not less than 6.5 h in the previous month before the experiment), the sleep–wake cycle was normal, and there was no shift history in 1 month; (2) no habit of drinking coffee, smoking, drinking alcohol, or drinking tea; (3) no recent history of acute infection, no history of hepatitis, tumor, nephritis, diabetes and endocrine disorders, etc; (4) no allergy history and no recent history of medication; and (5) no history of mental or neurological diseases. This study was approved by the Research Ethics Committee of the Academy of Military Medical Sciences. All participants signed the informed consent and were explained the procedure and compensated for their participation.

### Procedure

After passing the screening, all subjects were arranged the enrollment time and informed of them. During the experiment, they were uniformly managed, monitored, and escorted throughout the whole process. Subjects were not allowed to leave the laboratory and engage in intense activities. Food and drinks containing irritant and excitant substances were also forbidden. The experiment was designed as self-comparison and repeated measurements. All subjects got up at 6:00 a.m. on the day of the experiment, entered the experiment site at 7:00 a.m. and had breakfast, and began the sleep deprivation experiment at 8:00 a.m. Resting-state EEG was recorded twice, once during RW and once after 24 h of TSD.

The visual analogue scale (VAS) was used to obtain participants’ subjective ratings of their sleepiness. There are eight levels of sleepiness (1: fully awake, feeling active, and alert; 2: be able to concentrate, and all kinds of functions are at a high level, but not at the peak; 3: awake, relaxed, and responsive to external stimuli, but not fully awake; 4: a little bit confused and a little emotional; 5: confused, lost interest in staying awake, and slow to move; 6: sleepy, dizzy, trying to lie down, and trying to resist sleep; 7: no longer resisting sleep, quickly into sleep, and there are dreams in the mind; 8: fall asleep). The VAS was obtained twice: first, during RW and, second, after 24 h of TSD. For sleepiness levels, paired *t* test was performed between RW and TSD.

### EEG Acquisition

The whole EEG data collection was conducted in a noise-free and low-light laboratory, where the subjects sat in a comfortable chair directly in front of the 21-in. monitor, 60 cm away, with the center of the subject’s field of vision at the same level as the center of the monitor. Subjects were asked to remain silent and always focus on the green cross just below the centerline of the monitor to reduce the impact of blinking and eye movements on the EEG signals. The international standard 10–20 system electrode placement method was adopted to install the electrode, and the recording electrodes of 31 channels were FP1, FP2, F7, F8, F3, F4, Fz, FC5, FC6, FC1, FC2, T7, T8, C3, C4, Cz, TP9, TP10, CP5, CP6, CP1, CP2, P7, P8, P3, P4, Pz, POz, O1, O2, and Oz. Eye movements were monitored with an additional electrooculography (EOG) channel. The EEG recording was referenced to the bilateral mastoid line. The EEG data were recorded using a 32-channel (Brain Products GmbH, Germany) 10–20 acquisition system. The parameter settings are as follows: sampling frequency, 1,000 Hz; band-pass filter, 0.05–100 Hz; and scalp electrode contact resistance, less than 5,000 Ω. The EEG resting state was measured for 5 min. Only 24 subjects were subjected to the following analysis because three subjects fell asleep during the EEG scan. Follow-up signs of sleep (sleep spindles, slow rolling eye movements, and K-complexes) were found in the EEG of three sleepy subjects.

### Off-Line EEG Preprocessing

Off-line EEG analysis was done using EEGLAB (v. 2010b) (Delorme and Makeig, 2004) in MATLAB 2014 (The MathWorks). The EEG data were down-sampled from 1,000 to 125 Hz and bad channels were interpolated using the *pop_interp* function from EEGLAB. The data were applied to the fourth-order non-causal Butterworth filter for band-pass filtering at 1 and 40 Hz at half-power cutoff frequencies. Epochs containing muscle activities or other artifacts were excluded. The components related to eye movement and electrocardiogram (ECG) (Jung et al., 2000) were identified and eliminated by independent component analysis (Makeig et al., 1996). EEG recordings were re-referenced from common reference at the bilateral mastoid line to average reference.

### Microstate Analysis

Microstate analysis was performed using the academic software Cartool (Brunet et al., 2011). The calculation of the microstate classes followed a process described in the article of Michel and Koenig (2018). The optimally fitted microstate class templates were computed by running a two-step spatial clustering analysis based on the modified version of the traditional *k*-means algorithm (Pascual-Marqui et al., 1995). The terrain corresponding to the local maximum time point of the GFP (Lehmann and Skrandies, 1980) was selected for clustering analysis to improve the SNR (Koenig et al., 2002; Britz et al., 2010).

The microstate analysis was summarized into three steps. Firstly, the *k*-means clustering was repeated with *k* = 2, …, 20 at the individual level and the meta-standard approach (Kikuchi et al., 2007) was employed to define the optimal number of clusters, resulting in four to seven microstate classes for individual subjects. Then, the *k*-means clustering was also repeated with *k* = 2, …, 20 on the map generated by the first step clustering calculation across subjects (Murray et al., 2008; Tomescu et al., 2014; Gschwind et al., 2016) and the meta-standard approach (Kikuchi et al., 2007) was employed to determine the optimal number of clusters, resulting in six microstate classes at the group level. Secondly, in the microstate segmentation, the *k*-means algorithm was first used to cluster the EEG preprocessed data into six classes at the individual level, and then six template maps across participants were obtained by group clustering each participant’s representative microstates (Michel and Koenig, 2018). Thirdly, the microstate classes were fitted back into the individual EEG data to define the microstate in the temporal domain. The highest spatial correlation between the instantaneous scalp topography and each microstate class was used to label a microstate to the original data at each sampling point (Murray et al., 2008; Michel and Koenig, 2018). It was not considered that the spatial correlation of instantaneous scalp topography was less than 0.5. Then, minimum microstate duration was not required and the individual temporal parameters for each class were calculated (D’Croz-Baron et al., 2019). The mean duration is the average duration that a microstate class is continuously presented. The time coverage represents the mean proportion of time of one microstate class that is present across the analysis epochs. The frequency of occurrence represents the number of times a class repeats per second, and the global explained variance (GEV) is the sum of the explained variances of each microstate weighted by the GFP (D’Croz-Baron et al., 2019). For each microstate measure, paired *t* test was performed between the RW and TSD. Subsequently, these results were corrected for multiple comparisons by using the false discovery rate (FDR) method. Finally, we computed Spearman’s rank correlation coefficients between the significant microstate class parameters and subjective sleepiness.

The microstate syntax was assessed as follows: the transitions of microstate classes were considered as a Markov chain, which is a system that moves from one state to another by a finite number of states. These transition probabilities from one class to another were calculated from the segments (all consecutive time points of identical label stands for one segment). The transition probability from one class to another was calculated, which was the frequency of transitions from this class to all other classes, excluding itself. After normalization, the probability of each possible transition for each subject was obtained. Paired *t* test was performed for each pair of state transitions in RW and TSD to check whether there are significant differences in the transition patterns between the RW and TSD conditions. Subsequently, the statistical result of the microstate syntax was corrected for multiple comparisons by using the FDR method.

## Results

### EEG Microstate Analysis

**FIGURE 1:**
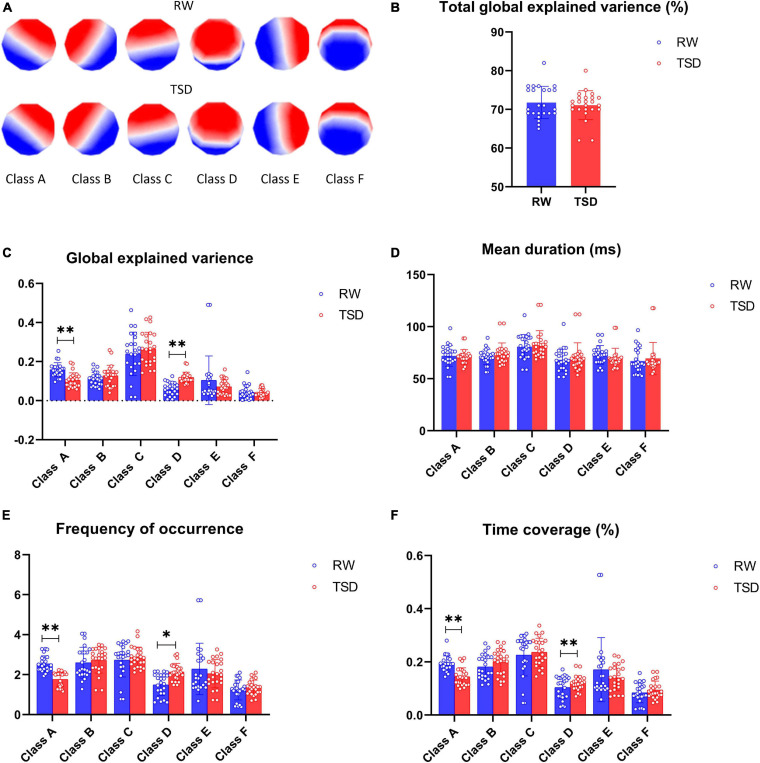
Microstate analysis results. **(A)** Spatial configuration of the six classes of microstates in rested wakefulness (RW) and total sleep deprivation (TSD). **(B)** The total global explained variance of all six microstates in RW and TSD. **(C)** Paired *t* test for the global explained variance revealed a significantly decreased class A and increased class D in TSD. **(D)** Paired *t* tests for the mean duration of classes A–F were not statistically significant in TSD compared with RW. **(E)** Paired *t* test for frequency of occurrence revealed a significantly decreased class A and increased class D in TSD. **(F)** Paired *t* test for time coverage revealed a significantly decreased class A and increased class D in TSD. *p < 0.05, paired *t* test; **p < 0.05, FDR corrected.

The six microstates across subjects in the RW and after 24 h of TSD explained about 70% of the global variance (71.7% in TSD and 70.9% in RW) (Figure 1B). Figure 1A shows the six microstate topographies, named classes A, B, C, D, E, and F, in RW and TSD that were consistent with previous literature reports (Custo et al., 2017; D’Croz-Baron et al., 2019). The temporal parameters for the six microstate classes are shown in Figures 1C–F.

The GEV varied between 0.06 and 0.49 in RW and TSD for the different microstate classes. Paired *t* test showed that the global explanatory variance of class A decreased significantly after 24-h TSD (*t* = 5.387, *p*-paired *t* test = 0.006, *p*-corrected = 0.036), but the global explanatory variance of class D increased significantly after 24-h TSD (*t* = -4.129, *p*-paired *t* test = 0.012, and *p*-corrected = 0.043) (Figure 1C).

For the different microstate classes, the microstate mean duration ranged between 69.54 and 87.32 ms in RW and TSD. Six microstate classes did not show statistically significant results regarding the parameters of microstate mean duration (Figure 1D).

The average number of occurrences per second for the different microstate classes ranged between 1.24 and 2.88 in RW and TSD. The frequency of occurrence of microstate class A was illustrated decreased in TSD, showing statistically significant differences (*t* = 7.653, *p*-paired *t* test = 0.003, and *p*-corrected = 0.027). However, microstate class D displayed a trend of increase in TSD compared to RW (*t* = -5.127, *p*-paired *t* test = 0.041, and *p*-corrected = 0.123) (Figure 1E).

The total percentage of time covered by the different microstate classes ranged between 12% and 28%. Class A showed a decrease in the ratio of time coverage after 24-h TSD, showing a statistically significant decrease (*t* = 9.322, *p*-paired *t* test = 0.002, and *p*-corrected = 0.027). It can be seen that class D became increased in the ratio of time coverage in the TSD condition, showing statistically significant differences (*t* = -4.517, *p*-paired *t* test = 0.011, and *p*-corrected = 0.043) (Figure 1F).

**FIGURE 2:**
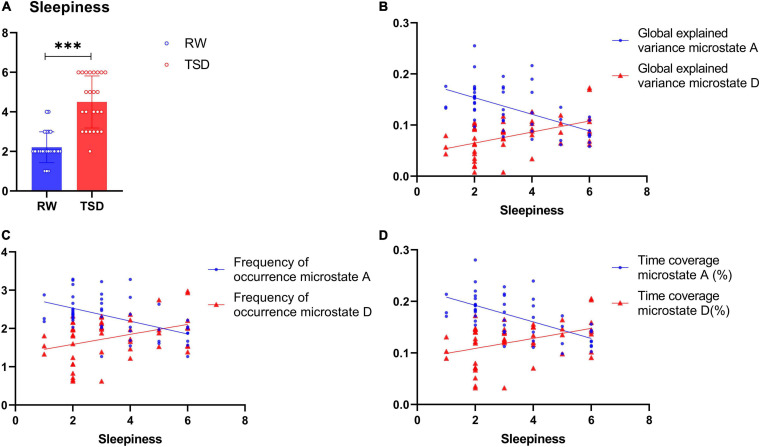
Spearman’s rank correlation between subjective sleepiness and microstate. **(A)** Levels of sleepiness in rested wakefulness (RW) and total sleep deprivation (TSD). **(B)** Regression plots of the correlation between sleepiness and the global explained variance (GEV) of class A (*r* = -0.567, *p* < 0.001) and class D (*r* = 0.481, *p* < 0.001), but significantly positively correlated with the GEV of class D. **(C)** Regression plots of the correlation between sleepiness and frequency of occurrence of class A (*r* = -0.516, *p* < 0.001) and class D (*r* = 0.367, *p* < 0.05), but significantly positively correlated with the frequency of occurrence of class D. **(D)** Regression plots of the correlation between sleepiness and time coverage of class A (*r* = -0.616, *p* < 0.001) and class D (*r* = 0.385, *p* < 0.01), but significantly positively correlated with the time coverage of class D. ***p < 0.001, paired *t* test.

In this study, the VAS was used to obtain participants’ subjective ratings of their sleepiness, yielding scores that range from 1 to 6. Paired *t* test showed that the sleepiness levels increased significantly after 24-h TSD (*t* = -13.89, *p* < 0.001) (Figure 2A). Spearman’s rank correlation between subjective sleepiness and microstate parameters showed that sleepiness was significantly negatively correlated with the GEV, frequency of occurrence, and time coverage of class A (GEV: *r* = -0.567, *p* < 0.001; frequency of occurrence: *r* = -0.516, *p* < 0.001; time coverage: *r* = -0.616, *p* < 0.001) and significantly positively correlated with the GEV, frequency of occurrence, and time coverage of class D (GEV: *r* = 0.481, *p* < 0.001; frequency of occurrence: *r* = 0.367, *p* < 0.05; time coverage: *r* = 0.385, *p* < 0.01) (Figures 2B–D).

### EEG Microstate Syntax

**FIGURE 3:**
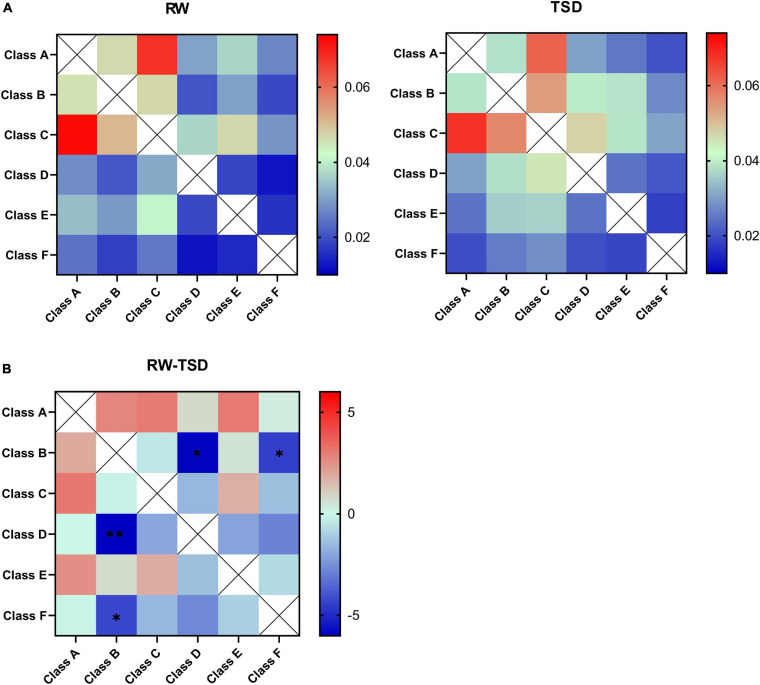
Syntax analysis results. **(A)** Probability transitions of the six classes of microstates in rested wakefulness (RW) and total sleep deprivation (TSD). **(B)** The *t* value was obtained by paired *t* test interaction for the state transition matrix. Differences between TSD individuals with respect to rest in the probability transition for each pair of state transitions show a significant increased probability of transition from class D to class B in TSD. *p < 0.05, paired *t* test; **p < 0.05, FDR corrected.

Figure 3A shows the probability transitions of the six classes of microstates in RW and TSD. Paired *t* test was performed for all double sequences of microstates between the RW and TSD conditions. In the syntax analysis, the probability of transition for one pair of state transitions displayed an increased presence in TSD compared to RW, which showed statistically significant differences, in D→B (*t* = -6.009, *p*-paired *t* test = 0.001, and *p*-corrected = 0.03) (Figure 3B). In addition, the probabilities of transition for three pairs of state transitions displayed a trend of increase in TSD, in B→D (*t* = -5.972, *p* = 0.004, and *p*-corrected = 0.06), B→F (*t* = -4.513, *p* = 0.009, and *p*-corrected = 0.072), and F→B (*t* = -4.012, *p* = 0.01, and *p*-corrected = 0.072) (Figure 3B).

## Discussion

In this study, we used microstate analysis to investigate the differences in the four temporal parameters and syntax of the dominant EEG microstate classes in 24 subjects between the RW and TSD conditions (with eyes open). The temporal characteristics analysis showed a significant decreased presence of class A and increased presence of class D in TSD compared to RW. Spearman’s rank correlation between subjective sleepiness and microstate parameters showed that sleepiness was significantly negatively correlated with appearances of class A and significantly positively correlated with appearances of class D. The syntax analysis revealed that class B exhibited a higher probability of transition than classes D and F in TSD compared to RW.

The combination of the two-step cluster analysis and meta-criteria produced six template topographies that best described the entire dataset and explained approximately 70% of the global variance. The first four of the six template topographies matched the canonical microstates (classes A–D) (Khanna et al., 2015; Michel and Koenig, 2018). The remaining two maps also corresponding to classes E and F were reported by Bréchet et al. (2019) and Custo et al. (2017). Therefore, the results showed that the EEG metrics obtained by the microstate analysis method had high repeatability.

In the present study, the presence of microstate class A was systematically lower in TSD compared to RW. We also found a significant increase in the time coverage and GEV of class D in TSD compared to RW. In some EEG–fMRI joint studies, it has been shown that class A was related to phonological processing (Damoiseaux et al., 2006; Mantini et al., 2007; Britz et al., 2010) in the resting state, which corresponded to the bilateral superior and middle temporal gyri (Britz et al., 2010). In previous studies on SD effect, it has been found that sleep deprivation seriously affected phonological information processing (Zhang et al., 2019). It was inferred that, consequently, TSD may lead to the decrease in the frequency of occurrence, GEV, and time coverage of class A in resting state. We also found robust effects, which were the significantly increased time coverage and GEV of class D in TSD compared to RW. The fourth microstate (class D) correlated with blood oxygenation level-dependent (BOLD) activity in the right-lateralized dorsal frontal and parietal cortices, which corresponded to the attention RSN (Britz et al., 2010). According to fMRI, sleep deprivation caused changes in the frontal and parietal areas (Goel et al., 2009). Previous studies found that activation of the prefrontal cortex, anterior cingulate gyrus, and thalamus (mainly involved in alertness, attention, and executive control) was decreased in 24-h TSD (Thomas et al., 2000; Choo et al., 2005). In this study, the subjects were required to keep their eyes open during the EEG recording. A possible explanation for the higher presence of class D after 24-h TSD was that more attention resource allocation may be needed to stay alert in the TSD condition. In addition, the parameters of microstate classes A and D were significantly correlated with the participants’ subjective ratings of their sleepiness, which seemed to be a good indication of individual differences of vulnerability to sleep deprivation (Yeo et al., 2015).

Many clinical evidences have shown that sleep deprivation is an effective treatment for major depressive disorder (MDD) (Giedke and Schwarzler, 2002; Bosch et al., 2013). EEG microstate research on MDD showed that the presence of class D was decreased in patients with MDD, which may indicate a decrease in cognitive control and ability to attend to environmental stimuli in MDD (Murphy et al., 2020). In addition, the increase of class A was associated with the severity of depressive symptoms, which may be associated with the abnormal processing of negative mood and cognition (Damborská et al., 2019). MDD and sleep deprivation had opposite effects on classes A and D, which may confirm that sleep deprivation had a role in combating and treating MDD symptoms (Bosch et al., 2013).

In the present study, we examined the microstate syntax in the RW and TSD conditions. We found that 24-h TSD induced a higher frequency of switching between states (B→D, D→B, B→F, and F→B). Previous studies have shown that the activation areas of class B were located in the primary visual cortex and right insular cortex (Mantini et al., 2007; Britz et al., 2010; Custo et al., 2017), and the insular has been thought to be a critical hub of the salience network (Menon and Uddin, 2010). Class D was shown to correlate with activations in areas corresponding to the attention network, including the right-lateralized dorsal frontal and parietal cortices (Mantini et al., 2007; Britz et al., 2010). It was assumed that the activation areas of class F corresponding to the DMN (Custo et al., 2017; Bréchet et al., 2019). Previous studies have suggested that abnormal interactions between SN, ECN, and DMN play an important role in the cognitive dysfunction of various mental diseases (Lerman et al., 2014; Zhang et al., 2017). After sleep deprivation, abnormal interactions between these networks were also reported by Lei et al. (2015). They proposed that alterations in SN–DMN coupling may be related to the cognitive alterations that underlie the lapse after TSD. These specific network interactions increased to meet the demands of a longer waking state. Extended wakefulness may accelerate the shifts between the dorsally generated RW-associated states and the centrally generated sleep-promoting ones (Doran et al., 2001; Goel et al., 2009). We boldly speculate that the higher frequency of transitions between classes D, F, and B indicated the competition between staying alert and falling asleep. This further indicated that the distribution of saliency in internal mental events was upregulated and that the transition between SN and attention network was also increased to meet the demands of a longer awake state. It should be noted that previous resting-state fMRI studies have reported decreases of transitions between dFC states following SD (Teng et al., 2019; Lombardo et al., 2020). One possible reason for this inconsistent finding is that the moving average dFC in fMRI analysis may have smoothened the rapid state transitions associated with wake-state instability (Teng et al., 2019).

In this study, we only assessed male volunteers, so we cannot make generalizations in females. Previous neuroimaging research has shown significantly higher activity in the left cerebellum posterior lobe, left parietal lobe, and bilateral frontal lobes after 24 h of TSD in males compared to females (Dai et al., 2012). In addition, it has been shown that males and females differ in the duration of the EEG microstate class C and the occurrence of class D in resting state (Tomescu et al., 2018). Therefore, sex-specific changes in microstate dynamics following TSD should be investigated in the future.

## Conclusion

In summary, using the microstate analysis method, robust changes in the temporal characteristics of specific brain states are detected after SD, such as frequency of occurrence, the GEV, and time coverage. Our findings suggest alterations of fast-changing, global neuroelectric patterns after total sleep deprivation in healthy young male subjects.

## Data Availability Statement

The datasets analyzed in the current study are available from the corresponding author upon reasonable request.

## Ethics Statement

This study was approved by the Research Ethics Committee of Academy of Military Medical Sciences. All participants signed the informed consent and explained the procedure and were compensated for their participation.

## Author Contributions

MK, JL, and LW designed the experiment and revised the manuscript. MK and JL wrote the manuscript. LW recorded and collected the data. JL performed the data analysis. All authors contributed to the article and approved the submitted version.

## Conflict of Interest

The authors declare that the research was conducted in the absence of any commercial or financial relationships that could be construed as a potential conflict of interest.
