# Supplementary material for: Alteration in Resting-State EEG Microstates Following 24 Hours of Total Sleep Deprivation in Healthy Young Male Subjects
Source: Front Hum Neurosci. 2021 Apr 12;15:636252. doi: 10.3389/fnhum.2021.636252 (PMC8075097; doi:10.3389/fnhum.2021.636252)
Supplement: Supplementary file 1 [file Data_Sheet_1.PDF]

Supplementary Material

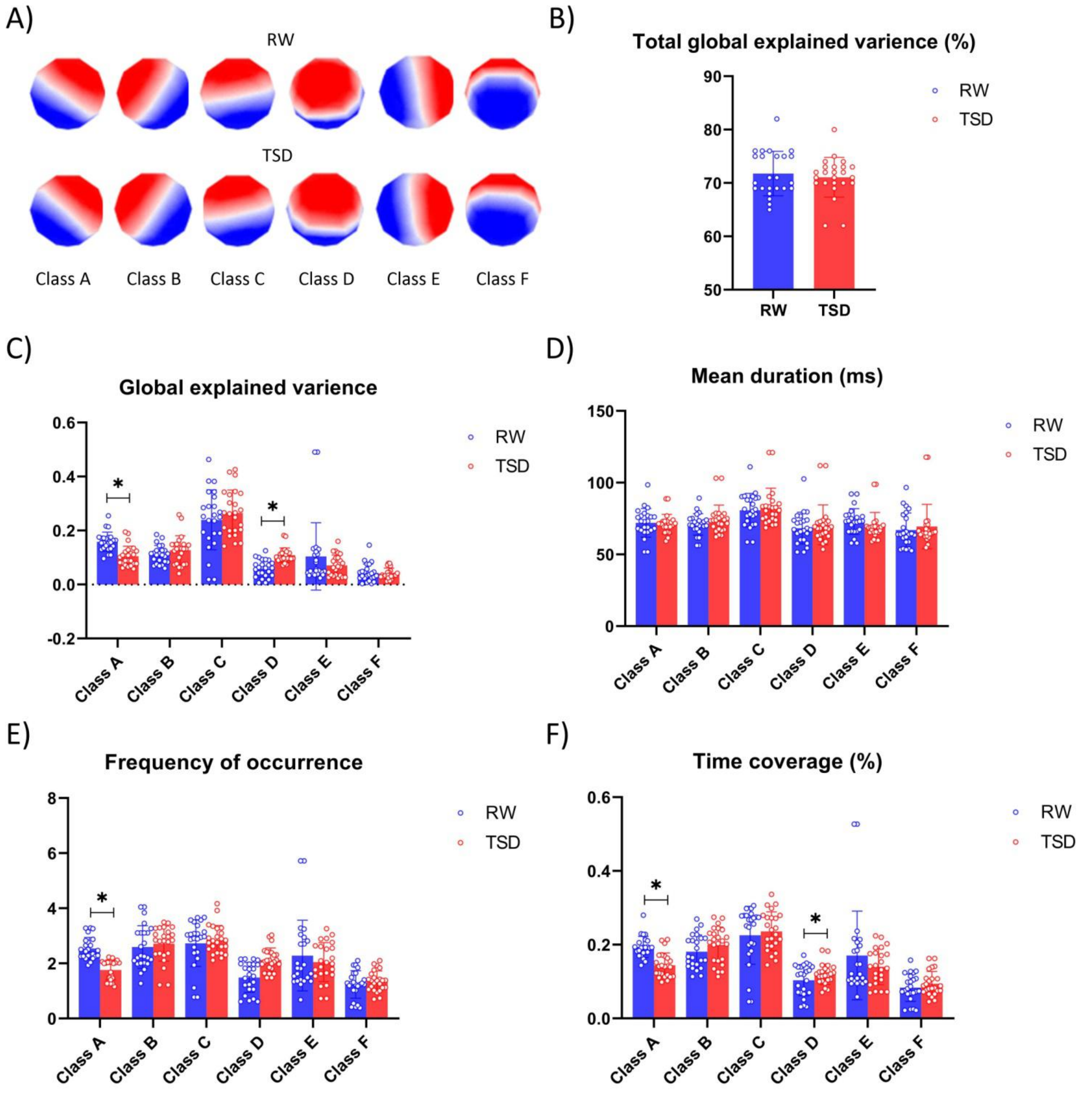

Figure 1: Microstate analysis results: (A) the spatial configuration of the six classes of microstates in RW and TSD; (B) The total global explained variance of all six microstates in RW and TSD; (C) paired t-test for Global Explained Variance revealed significantly decreased class A and increased class D in TSD; (D) paired t-test for mean duration class A-F were not statistically significant in TSD compared with RW; (E) paired t-test for frequency of occurrence revealed significantly decreased class A and increased class D in TSD; (F) paired t-test for time coverage revealed significantly decreased class A and increased class D in TSD. *: p<0.05, FDR corrected.

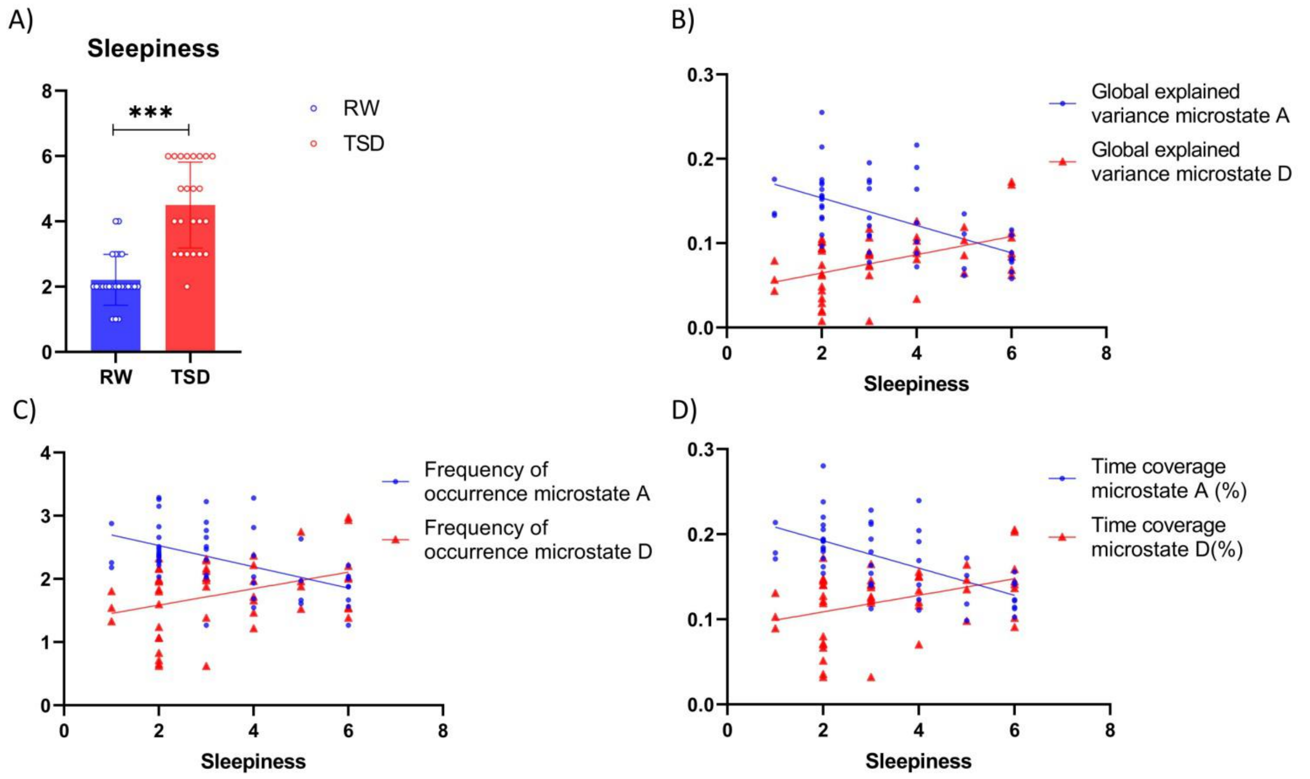

Figure 2: Spearman rank correlation between subjective sleepiness and microstate: (A)the levels of sleepiness in RW and TSD; (B)Regression plots of correlation between sleepiness and GEV of class A (r =-0.567, p<0.001)and class D (r =0.481, p<0.001), but significantly positively correlated with GEV of class D; (C) Regression plots of correlation between sleepiness and frequency of occurrence of class A (r=-0.516, p<0.001)and class D (r=0.367, p<0.05), but significantly positively correlated with frequency of occurrence of class D; (D)Regression plots of correlation between sleepiness and time coverage of class A (-0.616, p<0.001) and class D (r=0.385, p<0.01), but significantly positively correlated with time coverage of class D. ***: p<0.001, paired t-

test.

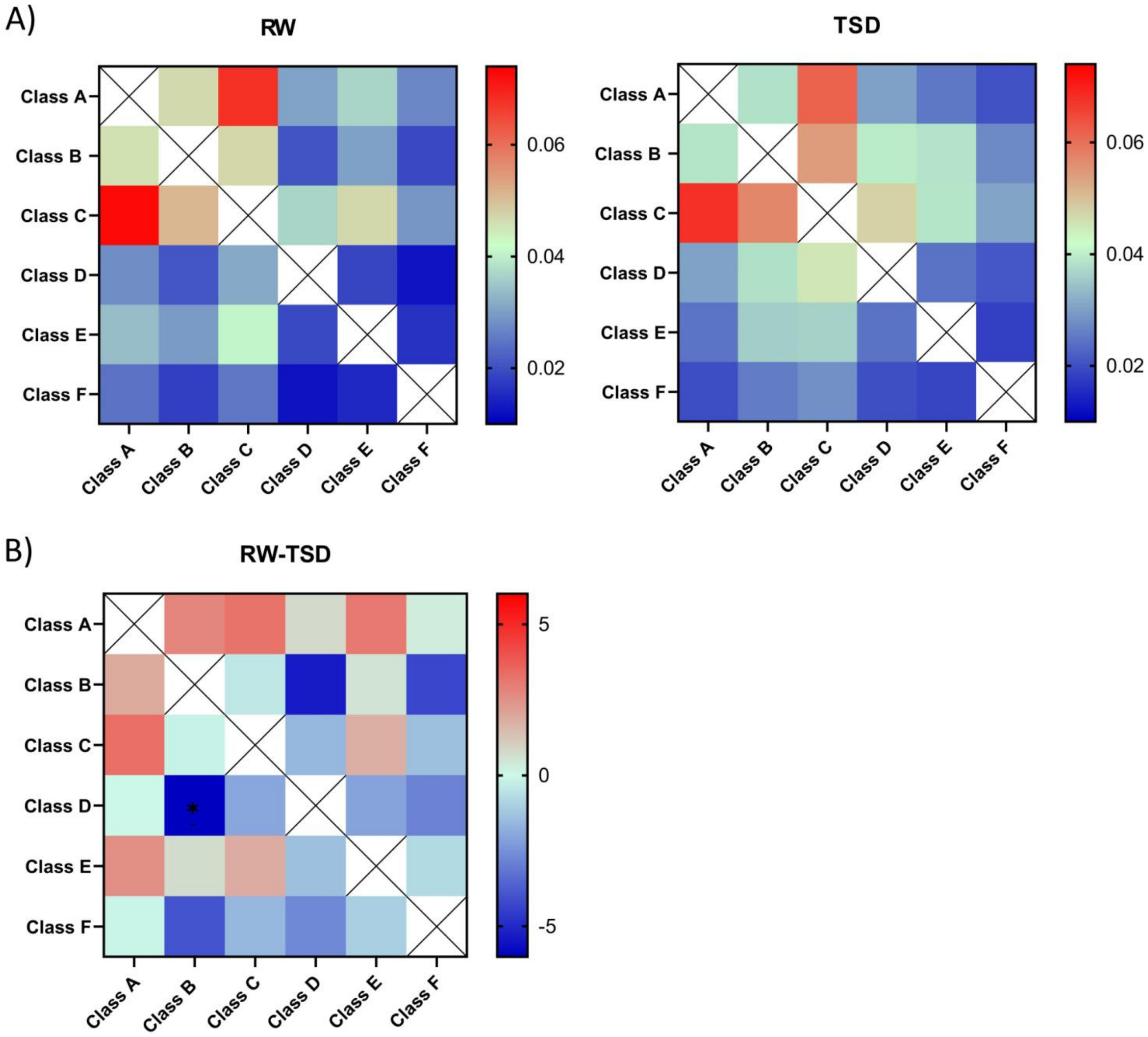

Figure 3: Syntax analysis results: (A) the probability transitions of the six classes of microstates in RW and TSD; (B) the t value was obtained by paired t-test interaction for the state transition matrix. Differences between TSD individuals with respect to rest in probability transition for each pair of state transitions, show significant increased probability of transition from class D to class B in TSD. *: p<0.05, FDR corrected.
